# Isolation and Identification of the Antimicrobial Agent Beauvericin from the Endophytic* Fusarium oxysporum* 5-19 with NMR and ESI-MS/MS

**DOI:** 10.1155/2016/1084670

**Published:** 2016-06-19

**Authors:** Huawei Zhang, Chuanfen Ruan, Xuelian Bai, Miao Zhang, Shuangshuang Zhu, Yingying Jiang

**Affiliations:** ^1^School of Pharmaceutical Sciences, Zhejiang University of Technology, Hangzhou 310014, China; ^2^Department of Life and Environmental Sciences, Hangzhou Normal University, Hangzhou 310036, China

## Abstract

Endophytic microbe has been proved to be one of rich sources of bioactive natural products with potential application for new drug and pesticide discovery. One cyclodepsipeptide, beauvericin, was firstly isolated from the fermentation broth of* Fusarium oxysporum* 5-19 endophytic on* Edgeworthia chrysantha* Linn. Its chemical structure was unambiguously identified by a combination of spectroscopic methods, such as HRESI-MS and ^1^H and ^13^C NMR. ESI-MS/MS was successfully used to elucidate the splitting decomposition route of the positive molecule ion of beauvericin. Antimicrobial results showed that this cyclodepsipeptide had inhibitory effect on three human pathogenic microbes,* Candida albicans*,* Escherichia coli*, and* Staphylococcus aureus*. In particular, beauvericin exhibited the strongest antimicrobial activity against* S. aureus* with MIC values of 3.91 *μ*M, which had similar effect with that of the positive control amoxicillin.

## 1. Introduction

**Figure 1:**
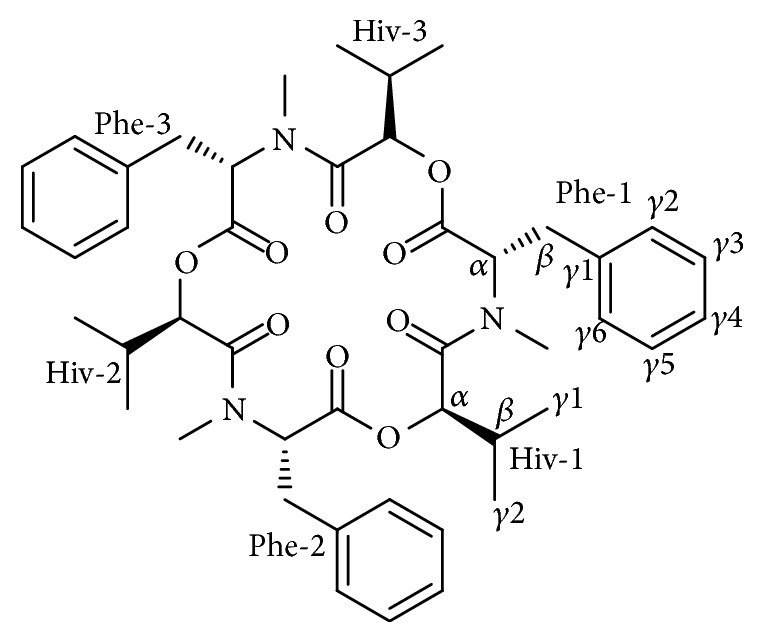
Chemical structure of beauvericin.

Bioactive natural products play a highly important role in the new drug and pesticide discovery [1, 2]. Beauvericin (Figure 1), a cyclic hexadepsipeptide with alternating* N*-methyl-phenylalanyl and* D*-hydroxy-isovaleryl residues, was firstly discovered from the entomopathogenic fungus* Beauveria bassiana* [3]. As one of chemical contaminants, it is mainly detected in maize kernels and derived products infected by phytopathogenic fungi, including* Aspergillus* [4],* Cordyceps* [5], and* Fusarium* [6–9] species. Originally, this cyclodepsipeptide was found to be toxic to human and animal tissues and cells [10]. However, many pharmacological studies suggested that it has a broad spectrum of biological activities, such as insecticidal [11], antimicrobial [12], antiviral [13], and antitumor [14]. Therefore, more attention has been paid to beauvericin as a new druggable chemical entity.

Endophyte, one of symbiotic microbes colonizing in healthy plants, has abundant biological diversity [15]. A growing evidence indicates that this special microorganism is one of rich sources of natural products with potent bioactivities, which have potential application in medicines and pesticides [15, 16]. In our previous antimicrobial screening of endophytic fungi from the healthy medicinal plant* Edgeworthia chrysantha* Lindl., the ethyl acetate extract of fermentation broth of a strain,* Fusarium oxysporum* 5-19, exhibited strong inhibitory effect on human pathogens [17]. A follow-up chemical investigation of this endophyte leads to the isolation of beauvericin with the yield of about 2.5 mg/L. Details of the isolation and structure elucidation of this cyclodepsipeptide with NMR and ESI-MS/MS were presented in this work as well as its antimicrobial activity. To date, it was the first report in which beauvericin was isolated from the fermentation broth of the endophytic* F. oxysporum* associated with* E. chrysantha* Lindl.

## 2. Materials and Methods

### 2.1. Strains

The endophytic strain 5-19 was isolated from the healthy medicinal plant* E. chrysantha* Lindl. collected at Zhaohui Campus of Zhejiang University of Technology (Hangzhou, China) and identified as* Fusarium oxysporum* on the basis of its morphological characteristics and 18S rDNA gene sequence (GenBank accession number KR019682). It has been transferred into potato dextrose agar (PDA) slants followed by storing at 4°C. Three human pathogenic strains,* Escherichia coli* AB 94012,* Staphylococcus aureus* AB 2010021, and* Candida albicans* AY 204006, were purchased from China Center for Type Culture Collection (CCTCC) and used as antimicrobial indicators.

### 2.2. General Experimental Procedures

Melting point was obtained on an X-4 digital display micromelting point apparatus without correction (Longtuo Instrument, Shanghai, China). UV spectrum was recorded on a UV-2450 UV/VIS spectrophotometer (Shimadzu, Japan). HRESI-MS spectrum was measured on an Agilent 6210 LC/TOF mass spectrometer (Agilent Technologies, CA, USA). ESI-MS/MS spectrum was carried out on a Finnigan LCQ Advantage Max ion trap mass spectrometer (Thermo Electron, CA, USA). ^1^H and ^13^C NMR spectra were performed on a Bruker Advance III spectrometer (Unity Plus 500 MHz) (Bruker, Switzerland). All solvents used in the study were of analytical grade.

### 2.3. Fermentation, Extraction, and Isolation

The producing strain 5-19 was cultured on PDA at 28°C for 7 days. One small piece of mycelium was inoculated aseptically to 250 mL Erlenmeyer flasks each containing 100 mL of PD liquid medium, and the seed liquids were incubated at 28°C for 3 days on a rotary shaker at 150 rpm. Then a balanced amount of fungal colony (10 mL) was transferred to culture broth in a 500 mL Erlenmeyer flask each containing 200 mL of Czapek medium consisting of glucose 30 g/L, NaNO_3_ 3 g/L, K_2_HPO_4_ 1 g/L, KCl 0.5 g/L, MgSO_4_·7H_2_O 0.5 g/L, and FeSO_4_ 0.01 g/L followed by shaking at 150 rpm at 28°C for 10 days. At the end of fermentation, all broth was collected and filtered through gauze, which afforded the filtrate (approximate 50 L) followed by extraction with the same volume of ethyl acetate (Merck). Then, the upper solvent was separated and evaporated at 25°C in vacuum to yield the extract (5.0 g).

The afforded extract was dissolved in methanol followed by filtration and subjected to fast separation on a HPLC apparatus (Water D600) equipped with a preparative column (Phenomenex Gemini-NX C18, 50 mm × 21.2 mm, 5 *μ*m) to give six fractions, F0–F5. Then, F4 (250.7 mg) was further subjected to HPLC with a semipreparative HPLC column (Phenomenex, Synergi Hydro-RP, 250 mm × 10 mm, 5 *μ*m) to afford beauvericin (125 mg) (Figure 1).

*Beauvericin*. White acicular crystals; m.p. 93-94°C; UV (MeOH) *λ*_max_ 210 nm; ESI-MS/MS (positive):* m/z* 806 [M + Na]^+^, 645, 545, 384; HRESI-MS (positive):* m/z* 806.4020 [M + Na]^+^ (calcd for C_45_H_57_N_3_NaO_9_, 806.3987); ^1^H and ^13^C NMR data were shown in Table 1.

### 2.4. Antimicrobial Assay

Antibacterial activity was assessed by the microbroth dilution method in 96-well culture plates [18]. The test compound was initially made up to 500 *μ*M in DMSO. Two commercial fungicides, amoxicillin and amphotericin B, were used as positive controls, and the solution of equal concentration of DMSO was used as a negative control. The tested fungi were incubated in the potato dextrose broth for 48 h at (28 ± 0.5)°C at 150 rpm, and the bacteria were cultured in the lysogeny broth for 24 h at (37 ± 0.5)°C at the same speed. Spores of different microorganism concentrations were diluted to approximately 1 × 10^6^ cfu with stroke-physiological saline solution (SPSS). All samples at 500 *μ*M (20 *μ*L) were added to 96-well microplates. Serial dilutions were made in the 96-well round-bottom sterile plates, and then the fungal suspension (40 *μ*L) and nutrient solution (40 *μ*L) were added. After incubation, minimum inhibitory concentration (MIC) was taken as the lowest concentration of the test compounds in the wells of the 96-well plate in which the lowest microbial growth could be measured at 600 nm. All tests were also carried out in triplicate.

## 3. Results and Discussion

The compound was obtained as white acicular crystals. Its HRESI-MS spectrum showed a quasi-molecular ion at* m/z* 806.4020 [M + Na]^+^ (calcd for C_45_H_57_N_3_NaO_9_, 806.3987), which possesses nineteen degrees of unsaturation. And the UV spectrum of this compound exhibited its maximum absorption peak at 210 nm. ^1^H NMR spectrum indicated that the chemical structure of this metabolite has three methyls [*δ*_H_ 0.39 (3H, d, *J* = 6.5 Hz), 0.80 (3H, d, *J* = 6.5 Hz), and 3.02 (3H, s)], methylene [*δ*_H_ 1.98 (2H, dd, *J* = 5, 15 Hz)], three methines [*δ*_H_ 1.97 (1H, m), 4.87 (1H, d, *J* = 8.5 Hz), and 5.57 (1H, d, *J* = 7.7 Hz)], and a benzene ring [*δ*_H_ 7.28 (5H, ar)]. The ^13^C NMR spectrum in combination with the analysis of the DEPT and HSQC spectra also suggested the presence of fifteen carbon signals, including two carbonyls, three methines, one methylene, three methyls, and a benzene ring. It was supposed that this compound consists of three identical moieties, each of which has fifteen carbons. By careful inspection of its ^1^H and ^13^C NMR spectral data (Table 1), which were identical with those of beauvericin reported in literature [3, 19], this compound was ambiguously characterized.

**Figure 2:**
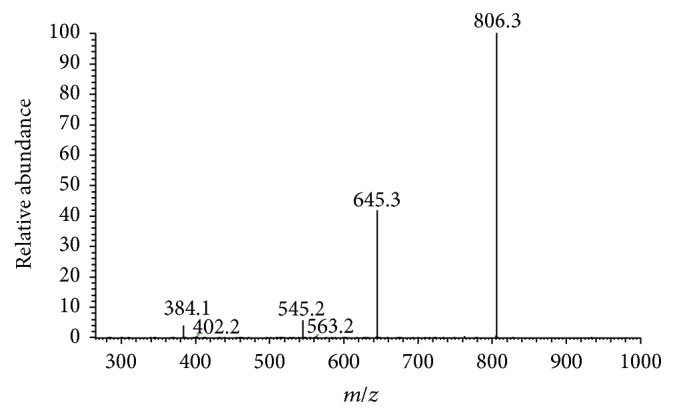
ESI-MS/MS spectrum of beauvericin.

**Figure 3:**
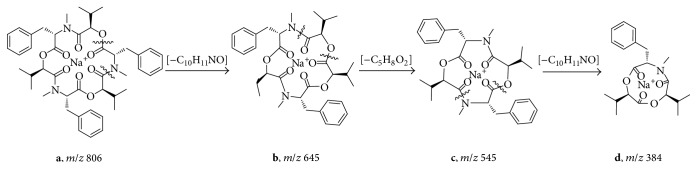
The splitting decomposition route of the molecule ion of beauvericin.

Beauvericin was cyclohexadepsipeptide composed of three units of phenylalanine (Phe) and three units of 2-hydroxyisovaleric acid (Hiv). The ESI-MS/MS spectrum shown in Figures 2 and 3 further reinforced the chemical elucidation of beauvericin, which the splitting decomposition route clearly explained. Initially, the positive parent-molecule ion of beauvericin** a** (*m/z* 806) was changed into** b** (*m/z* 645) by removing a fragment [C_10_H_11_NO]. Then, the daughter ion** c** (*m/z* 545) formed through separating a fragment [C_5_H_8_O_2_] from** b**. Further removal of anther fragment [C_10_H_11_NO] from** c** yielded the final ion** d**.

Antimicrobial assay indicated that beauvericin had inhibitory effect on all testing strains,* C. albicans*,* E. coli*, and* S. aureus* (Table 2). In particular, this cyclodepsipeptide exhibited the strongest antimicrobial activity against* S. aureus* with MIC value of 3.91 *μ*M, which was similar to that of the positive control amoxicillin.

## 4. Conclusions

Recently, the fermentation conditions of beauvericin production had been optimized in large scale for application in pesticide and medicine industry [20, 21]. In the present study, chemical investigation of the endophytic fungus* F. oxysporum* 5-19 associated with* E. chrysantha* Linn. leads to the isolation of beauvericin, in which the yield was approximately 2.5 mg/L. Its chemical structure was unambiguously identified by a combination of spectroscopic methods, including HRESI-MS and ^1^H and ^13^C NMR. ESI-MS/MS was successfully used to elucidate the chemical structure of beauvericin. To date, it was the first report in which beauvericin was isolated from the fermentation broth of endophytic* F. oxysporum* 5-19 and found to have potent inhibitory effect on the growth of pathogenic* S. aureus* with the MIC value of 3.91 *μ*M. Therefore, beauvericin could be produced in industrial level and directly used as the raw material for development as a new therapeutic agent for treatment of the infection disease caused by the pathogenic* S. aureus*.

## Figures and Tables

**Table 1:** ^1^H and ^13^C NMR data for beauvericin in CDCl_3_ at 500 MHz.

Position	*δ*_C_	*δ*_H_ (*J* in Hz)
*Phe*		
CO	170.0	—
*α*	75.6	4.87 (1H, d, 8.5 Hz)
*β*	34.69	1.98 (2H, dd, 5, 15 Hz)
*γ*1	126.79	—
*γ*2	128.54	7.18 (1H, t)
*γ*3	128.78	7.26 (1H, d, 10 Hz)
*γ*4	136.53	7.23 (1H, dd, 10, 20 Hz)
*γ*5	128.78	7.26 (1H, d, 10 Hz)
*γ*6	128.54	7.18 (1H, t)
N–CH_3_	32.13	3.02 (3H, s)
*Hiv*		
CO	169.8	—
*α*	57.1	5.57 (1H, d, 7.7 Hz)
*β*	29.7	1.97 (1H, m)
*γ*1	17.3	0.39 (3H, d, 6.5 Hz)
*γ*2	18.3	0.80 (3H, d, 6.5 Hz)

**Table 2:** MIC values of beauvericin against three human pathogenic microbes (*μ*M).

Compound	*E. coli*	*S. aureus*	*C. albicans*
Beauvericin	62.5	3.91	>250
Amoxicillin	4.76	2.38	—
Amphotericin B	—	—	2.1
DMSO	—	—	—

Ampicillin and amphotericin B were positive controls.

—: not evaluated.
